# Pharmarcomechanical thrombectomy combined with transluminal balloon angioplasty for treating transplant renal vein thrombosis

**DOI:** 10.1038/s41598-023-44514-8

**Published:** 2023-10-12

**Authors:** Shao-Jie Wu, Chi Zhang, Min Wu, Dan-dan Ruan, Yan-ping Zhang, Bin Lin, Yi Tang, Xin Chen, Chen Wang, Hong-hong Pan, Qing-guo Zhu, Jie-wei Luo, Lie-fu Ye, Zhu-ting Fang

**Affiliations:** 1 Fujian Provincial Hospital, Shengli Clinical Medical College of Fujian Medical University, Fuzhou, 350001 China; 2 Department of Interventional Radiology, Fujian Provincial Hospital, Fuzhou, 350001 China; 3 Department of Urology, Fujian Provincial Hospital, Fuzhou, 350001 China; 4 Pathology Department, Fujian Provincial Hospital, Fuzhou, 350001 China; 5 Department of Traditional Chinese Medicine, Fujian Provincial Hospital, Fuzhou, 350001 China

**Keywords:** Nephrology, Urology

## Abstract

Renal vein thrombosis (RVT) is a rare vascular complication that occurs after renal transplantation and usually results in irreversible kidney damage and graft loss. We report the case of a patient who underwent right iliac fossa allogeneic kidney transplantation and developed RVT combined with ipsilateral thrombosis from the popliteal to the femoral veins, with extension to the common iliac veins, 4 months after transplantation. Under unfractionated heparin anticoagulation, an Aegisy (Life Tech Scientific Co., Ltd., Shenzhen, China) vena cava filter was placed to prevent pulmonary embolism. Percutaneous mechanical thrombectomy combined with balloon angioplasty was performed to aspirate the thrombus and successfully dilate the narrow venous lumen. The patient’s renal function was restored postoperatively. Ultrasonography showed the allograft and ipsilateral lower extremity deep veins to be fluent and patent. To conclude, in patients with RVT after renal transplantation, percutaneous mechanical thrombectomy in conjunction with balloon angioplasty can be performed with desirable outcomes and no severe adverse effects. This method reduces the risk of bleeding from exposure to systemic intravenous thrombolysis and avoids surgery-associated trauma.

## Introduction

Kidney transplantation is an effective treatment for end-stage renal disease, with allogeneic kidney transplantation being the most common form. Transplant-related complications are essential factors that influence the survival of transplanted kidneys^1^. Transplant renal vein thrombosis (TRVT) is a rare post-transplant complication with a reported prevalence of 0.1–4.2%^1,2^, and it remains a remarkable cause of graft loss and nephrectomy. Timely and effective intervention can save the allograft and improve patients’ quality of life. In February 2022, a case of transplanted renal vein and ipsilateral lower limb deep vein thrombosis (DVT) was diagnosed. The patient was successfully treated using emergency percutaneous Pharmarcomechanical (AngioJet (Boston Scientific, Marlborough, Massachusetts, USA) mechanical thrombectomy and transluminal balloon angioplasty, and an inferior vena cava (IVC) filter was placed beforehand to prevent pulmonary embolism. Pharmacomechanical devices have been directly used in the treatment of renal vein thrombosis (RVT), although reports are limited and rarely involve allograft^3^. The purpose of this article is to support Pharmarcomechanical mechanical thrombectomy and balloon angioplasty coupled with anticoagulation as a successful treatment for TRVT.

## Case presentation

The patient was a 42-year-old man with a body mass index of 21.9 kg/m^2^ and 13-year history of hypertension. He had started regular peritoneal dialysis treatment 3 years prior for chronic renal failure [chronic kidney disease (CKD), stage 5, uremic phase] caused by glomerulonephritis. Before renal transplantation, the patient was anuric, with a blood creatinine level of 1526 μmol/L and urea nitrogen of 21.6 mmol/L. There was no history of malignancy, vascular disease, other kidney diseases, or other diseases with similar characteristics. The laboratory results for coagulation factors were unremarkable.

On October 25, 2021, the patient accepted an allogeneic kidney transplant from a 27-year-old male donor who was diagnosed as brain death following a car accident. The donor’s left kidney was transplanted to the recipient’s right iliac fossa. The donor’s renal artery and vein were anastomosed to those of the recipient, both by end-to-side. From the 3rd day following the operation, urine output was established as standard and observed to be approximately 2000 mL per day, with a urea nitrogen concentration of 12.2 mmol/L (normal reference level: 3.2–7.1 mmol/L) and creatinine of 136 μmol/L (normal reference level: 44–132 μmol/L) 1 week postoperatively; these levels returned to their normal ranges at the subsequent follow-up. Multiple ultrasound examinations were performed within a week after surgery and showed normal arterial and venous flow in the allograft. Triple immunosuppressive therapy with tacrolimus, mycophenolate mofetil, and methylprednisolone was the antirejection regimen. The patient’s urine output was typical during the next 4-month follow-up; however, the creatinine level gradually increased to 264 µmol/L. The allograft artery showed mildly elevated resistance index (RI = 0.82) on ultrasonography; however, there was no evidence of substantial thrombosis in the arteries or veins of the allograft. Ultrasonographically localized puncture biopsy was performed on February 25, 2022. Pathological findings were acute T cell-mediated rejection (Banff grade IA), indicating an acute rejection, and mild–moderate dwarfism of the tubular epithelium, suggestive of renal tissue ischemia. The human leukocyte antigen (HLA)-classified antibody report showed that the mean fluorescence intensity (MFI) was moderate and weakly positive in the newly added de novo DSA to different epitopes of the donor’s HLA class II DQ2. The combined diagnosis was acute T-cell- and antibody-mediated rejection. In addition to the usual antirejection regimen, the treatment course included the use of steroid hormones, plasma replacement, and infusion of human intravenous immunoglobulin (IVIG), rabbit antihuman thymocyte globulin, and rituximab.

The patient experienced abrupt nausea and vomiting on the 3rd day of plasma exchange therapy (4 months after kidney transplantation) along with swelling and pain around the site of the transplanted kidney; this was followed by progressive swelling of the right lower leg. The circumference of the thigh at 15 cm from the upper edge of the patella was approximately 5 cm longer than that on the left side. The patient’s urine output was only 600 mL/24 h, which was much lower than the average of 1500–2000 mL/24 h. On examination, the right iliac fossa was found to be significantly mainly around the thigh. The urea concentration was 16.4 mmol/L and that for creatinine was 255 μmol/L, which were significantly higher than the previous day’s levels (urea nitrogen, 12.3 mmol/L; creatinine, 154 μmol/L). According to laboratory tests, prothrombin time (PT) and activated partial thromboplastin time (APTT) were within normal ranges, and the D-dimer concentration was 20 mg/L.

### Investigations

**Figure 1:**
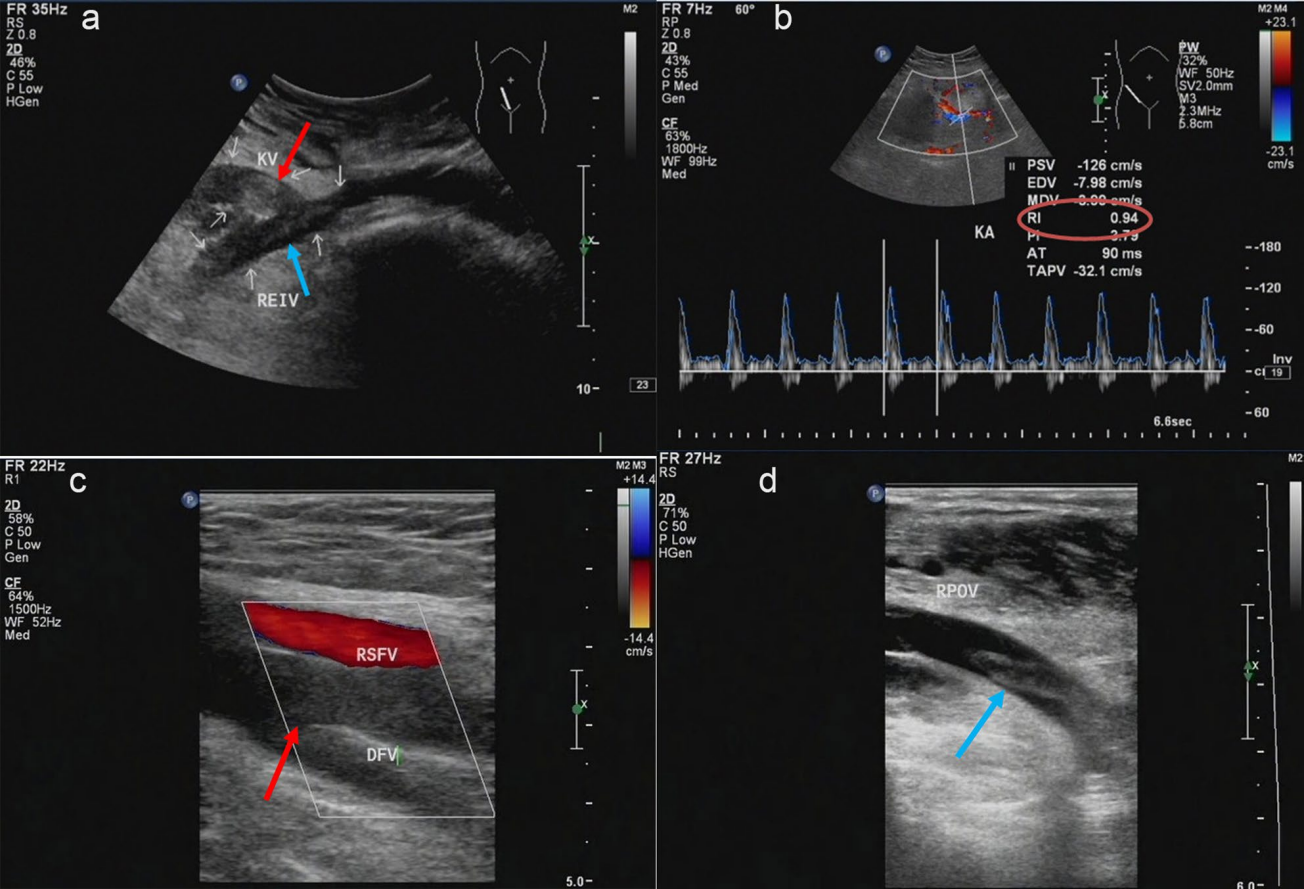
(**a**) An emergency Doppler ultrasound examination showed an anastomosis of thevein of the transplanted kidney to the right external iliac vein, with a widening of the renal vein and a heterogeneous hypoechoic filling of the lumen (Red arrow shows thrombosis of the main vein of the transplanted kidney), extending into the right external iliac vein (Blue arrow shows right superficial iliac vein thrombosis). (**b**) There was no significant flow in the renal vein or the right external iliac vein on Doppler ultrasound and the transplanted renal artery showed high resistance to flow with a resistance index (RI) of 0.94, which was significantly higher than normal and the patient’s previous baseline level. The intrarenal vein is slightly widened, with poor internal sound transmission and stagnant blood flow. Doppler ultrasound showed heterogeneous hypoechoic filling of the lumen of most segments of the deep veins of the right lower limb, with no significant blood flow signal. The rest of the segmental lumen is poorly permeable and the blood flow is stagnant. (**c**) Right femoral deep vein thrombosis shown by red arrow, no blood flow signal seen. (**d**) Partial thrombus filling of the lumen of the right posterior tibial vein shown by the blue arrow.

**Figure 2:**
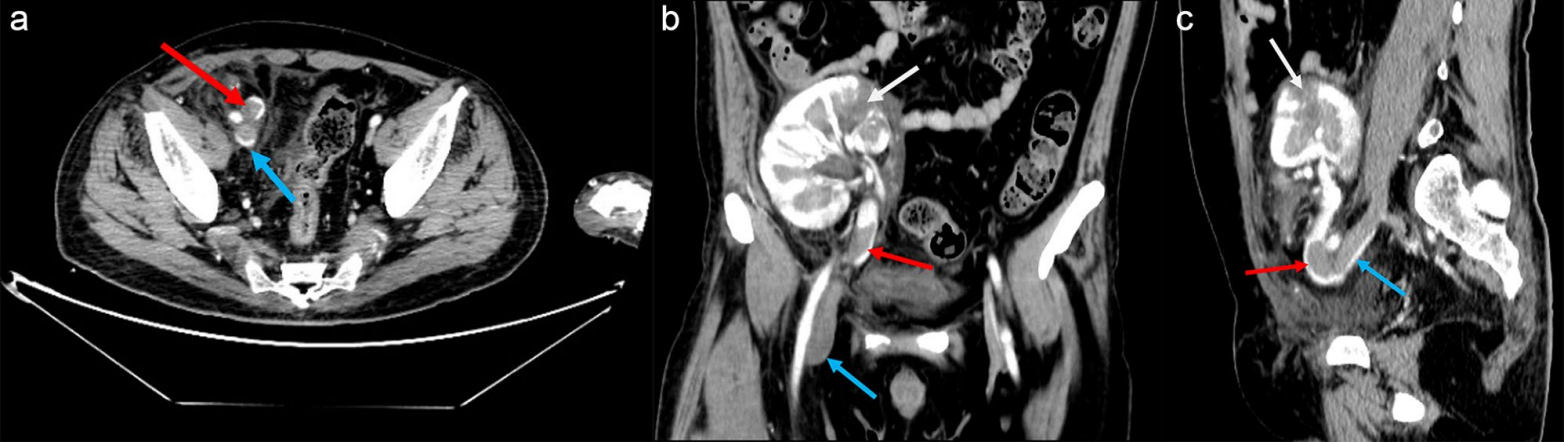
(**a**) Transverse section, (**b**) Coronal section, (**c**) Median sagittal. Emergency computed tomography shows swelling of the right iliac fossa graft kidney with varying renal cortical density, with patchy isoslightly hypodense foci, which are relatively hypodense on enhancement, partially indistinct, and with the tip directed towards the renal hilum; renal infarction was considered (indicated by white arrows). A filling defect is seen in the grafted right renal vein and is thrombosed (indicated by a red arrow). Filling defects in the right common iliac vein, the right internal and external iliac veins, the ingested portion of the right femoral vein, and the grafted renal vein is seen to be thrombosed (Blue arrow shows right superficial iliac vein thrombosis, median sagittal section shows continuity with right central renal vein thrombosis). Perirenal and adjacent pelvic fatty interstitial exudates and effusions.

The patient underwent ultrasound examination, which revealed that the right external iliac anastomosis, allograft veins were all thrombosed (Fig. 1a, b), and allograft artery elevated resistance index (RI = 0.94). Additionally, the deep veins of the right lower extremity, including the common femoral, deep femoral, popliteal, anterior tibial, posterior tibial, and peroneal veins, were filled with an inhomogeneous hypoechogenicity (Fig. 1c, d). Contrast-enhanced computed tomography (CT) confirmed thrombosis in the main vein of the transplanted kidney that extended to the right lower extremity veins (occlusive thrombus from the popliteal through to the femoral veins, with extension to the common iliac veins) (Fig. 2). Acute TRVT combined with ipsilateral lower-extremity DVT were confirmed.

### Treatment

**Figure 3:**
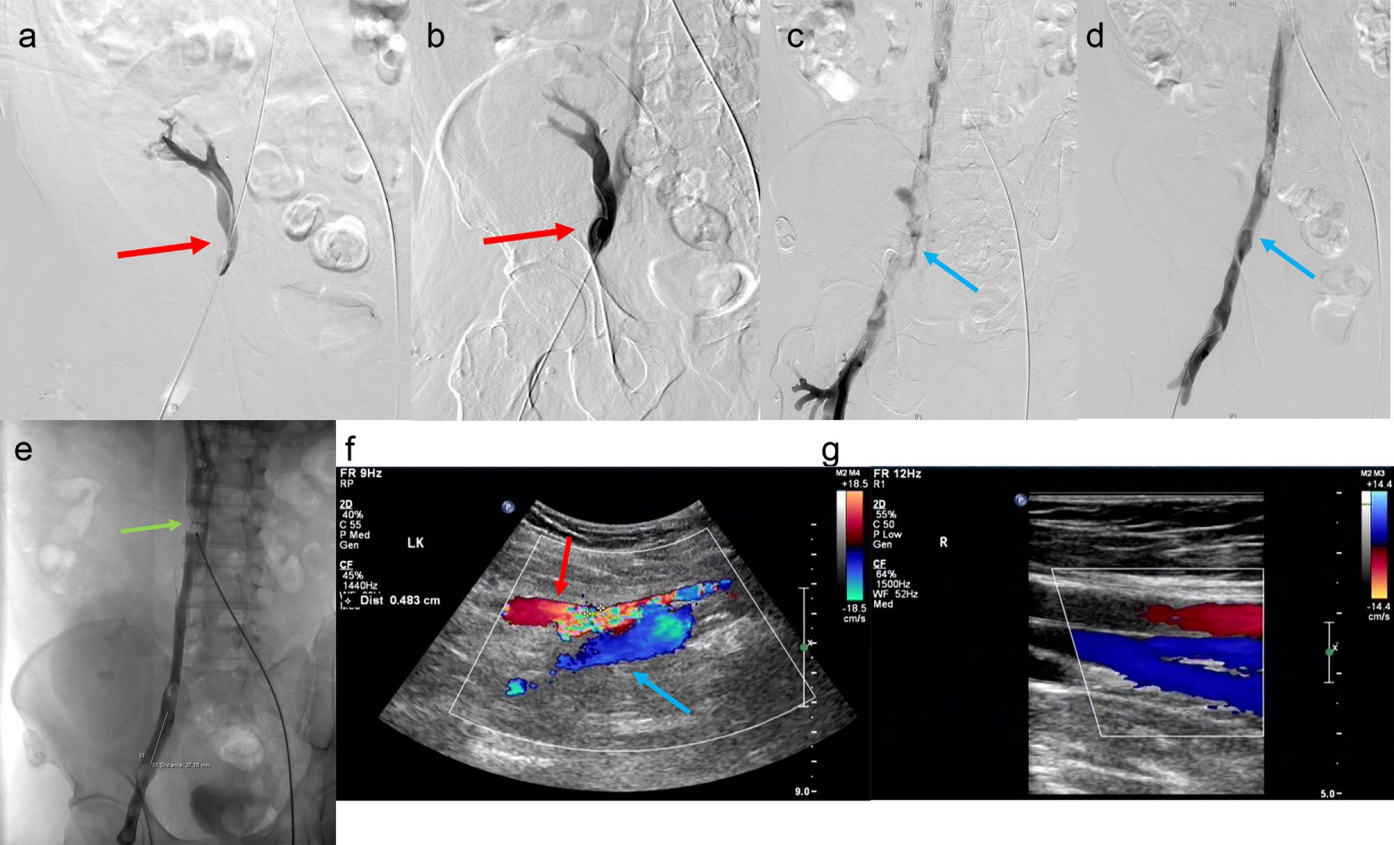
(**a**)–(**e**): DSA post-anterior view showing filling defects in the proximal main vein of the transplanted kidney and the right popliteal vein, femoral vein, common femoral vein, external iliac vein and common iliac vein. (**a**): The red arrow shows a limited filling defect at the confluence of the vein of the transplanted kidney into the right external iliac vein. (**b**): The lumen of the original vascular filling defect in the vein of the transplanted kidney, shown by the red arrow, is open and restored to its normal lumen diameter. Pharmarcomechanical mechanical thrombectomy plus transluminal balloon angioplasty after post-DSA contrast angiography shows a significant improvement in the lumen of the former limited filling defect in the vein of the transplanted kidney after treatment, with contrast filling. (**c**): Blue arrows show contrast filling defects and stagnant flow in the lumen of the right external iliac vein and common iliac vein. (**d**): Patency of the right external iliac vein and common iliac vein lumen, as indicated by the blue arrows, with thrombus clearance and smooth contrast reflux. The original right lower limb DVT-filled vessel lumen was largely cleared and the vessel lumen was restored to patency with smooth contrast return, no contrast stagnation, reflux or collateral vessel formation was observed. (**e**): Free thrombus captured underneath the pre-positioned inferior vena cava filter shown by the green arrow. Partial thrombus dislodgement during Pharmarcomechanical mechanical thrombectomy plus transluminal balloon angioplasty, DSA angiography shows a limited filling defect in the lumen of the inferior vena cava below the filter as a dislodged thrombus. (**f**)–(**g**): Doppler ultrasound 6 months after surgery. (**f**): Six months after the operation, Doppler ultrasound showed that the transplanted kidney could be detected in the right iliac fossa, with normal kidney morphology, parenchymal echogenicity, and no significant abnormalities in the renal sinuses. End-lateral anastomosis of the renal artery to the right external iliac artery, average renal artery resistance index, RI: 0.82, end-lateral anastomosis of the renal vein to the right external iliac vein. No significant abnormalities in renal artery and renal vein blood flow were noted. (The lumen of the aortic vessel of the transplanted kidney is patent, as indicated by the red arrow) (The lumen of the central vein of the transplanted kidney is patent, as indicated by the blue arrow). (**g**): The right common femoral, femoral, deep femoral, anterior tibial, posterior tibial, and popliteal veins are normal in diameter, with good intraventricular sound, unobstructed flow, and average flow spectrum pattern.

As soon as the diagnosis was made, continued perfusion of unfractionated heparin was performed to maintain an APTT of 60–80 s. Subsequently, the patient underwent interventional therapy in the angiography room. AngioJet (Boston Scientific, Marlborough, Massachusetts, USA) pharmacomechanical thrombectomy and transluminal balloon angioplasty were immediately performed. The process was as follows: the sheath was inserted into the left femoral vein using Seldinger’s method. Cavography was performed routinely in the anteroposterior and lateral positions using a 5-F pigtail catheter (Cordis, Tipperary, Ireland) to confirm the absence of thrombosis in the IVC and access veins. An Aegisy vena cava filter (LifeTech Scientific Co. Ltd, Shenzhen, China) was then placed in the IVC to prevent pulmonary embolism. Ultrasound-guided sheathing of the right popliteal vein and 5-F pigtail catheterization revealed filling defects in the allograft, anastomosis, and right lower extremity veins (comparable to the CT findings) with collateral circulation and contrast reflux. A 0.035-inch guidewire (Radifocus®; Terumo, Tokyo, Japan) fed into a 5-F VER catheter (Cordis, Tipperary, Ireland) was introduced from the right popliteal vein, into the allograft vein, and into the IVC through the thrombus. The Amplatz Fixed Core Wire Guide (Boston Scientific) was introduced to guide the AngioJet Thrombectomy catheter Solent™ Omni (Boston Scientific) to the thrombus lumen. Local injection thrombolysis mode (PowerPulse™ Delivery) was used. Afterward, 250 mL of 0.9% NaCl solution containing 200,000 units of urokinase was injected into the thrombus. Urokinase was retained for 30 min, and then the rheological suction mode for thrombus aspiration was implemented^4^. Following the aforementioned procedures, angiography showed a dislodged thrombus captured below the IVC filter. Thrombus residue occupied the venous lumen causing segmental narrowing in the veins of the allograft and the right lower extremity, including the anastomosis. Balloon (Boston Scientific, Marlborough, Massachusetts, USA) angioplasty was performed until 12 atm for 3 min followed by repeated aspiration venous thrombectomy, and total blood loss was controlled within 200 mL. The final phlebography showed patency with excellent flow in allograft, anastomosis, and the ipsilateral lower extremities (Fig. 3a–e). Iodixanol (150 mL; 32 g iodine per 100 mL) was used during the procedure.

The patient received (cefoperazone/sulbactam) administration for 3 days after the procedure. Low-molecular-weight heparin was used as an in-hospital anticoagulation regimen, and oral rivaroxaban was administered for 3 months after discharge. His primary antirejection therapy (tacrolimus and mycophenolate mofetil in combination with methylprednisolone) was initiated on postoperative day 3, and the IVC filter was removed 12 days postoperatively.

### Outcome and follow-up

PT, APTT, D-dimer, creatinine, urea nitrogen, and renal ultrasound were monitored daily for 3 days postoperatively. Creatinine, urea nitrogen, and blood levels of antirejection drugs were monitored biweekly. Within a week of hospitalization, the right iliac fossa pain was relieved, the circumference of the right thigh which was approximately 5 cm longer than the left previous returned to its normal, urine volume and kidney function gradually returned to baseline levels as showed in Table 1. Arterial resistance index before surgery was 0.94. Ultrasound showed allograft vein patency accompanied with artery resistance index (RI = 0.68) decrease at discharge. At 1, 3, and 6 months after the procedure, ultrasonography revealed an allograft artery resistance index (RI = 0.63–0.70) within the normal limits, along with good patency and excellent flow in veins of the allograft and right lower extremity (Fig. 3f, g). No serious complications, such as pulmonary embolism, vascular damage, or hemorrhage were observed.

**Table 1:** Creatinine, urea nitrogen, urine output changes.

Day/d	1 (Day before surgery)	2	3	4	5	6	7
Creatinine (μmol/L)	255	152	134	100	87	85	81
Urea nitrogen (mmol/L)	16.4	10.7	7.5	6	5.8	5.5	5.2
Urine output (mL/24 h)	600	1500	1600	1700	1600	1800	1700

## Discussion

Although kidney transplantation is the most common form of organ transplantation, complications are still unavoidable. In most cases, TRVT may occur locally in the graft vein, but may extend to the ipsilateral lower limb deep vein. In rare cases, the DVT may also extend proximally, leading to anastomosis obstruction and TRVT. In our case, the patient presented with swelling, pain in the transplanted kidney area, and oliguria, followed by progressive swelling of the right lower limb. The patient was diagnosed with acute rejection, possessed high-risk factors of thrombosis for allograft, overlap his basic diseases, and comprehensive antirejection treatment was initiated. It was presumed that venous thrombosis of the transplanted kidney extended to the ipsilateral lower limb.

The factors that trigger TRVT are diverse, but include donor and recipient factors, mechanical factors, rejection reaction, and immunosuppression^1,5^. Donor kidneys from deceased or aged donors and those which underwent prolonged isolation increase the risk of TRVT. These elements may extend ischemia in the allograft, which may damage the endothelium and cause cellular edema and thrombosis^6^. Recipient age, underlying diseases, and pretransplant dialysis modality are further risk factors for TRVT. More so, chronic conditions, such as old age, hypertension, diabetes, and atherosclerosis make vessels more susceptible to thrombosis. It has been shown that peritoneal dialysis is more likely to induce elevated plasma procoagulants and blood hypercoagulability than hemodialysis. Furthermore, membranous nephropathy may induce a corresponding immune response, thereby increasing the incidence of TRVT^7–9^. Mechanical factors, such as excessive length, twisting, compression of the grafted renal vein, constriction of the anastomosis, and the angle at which the grafted renal vein meets the iliac vein, were the most common causes^10,11^. During transplantation, the right kidney enters the left iliac fossa, and the left kidney enters the right iliac fossa; this transition can easily lead to compression of the renal vein by the renal artery^1,9^. Prolonged or high-dose administration of immunosuppressive drugs, such as cyclosporine, methylprednisolone, and antithymocyte/antilymphocyte globulin can increase the risk of graft thrombosis by inducing platelet aggregation, promoting thrombin production, and reducing fibrinolysis^1,12,13^. Our patient had a combination of these high-risk factors: first, the donor’s kidney was sourced from a brain-dead patient; second, the recipient was hypertensive and on long-term peritoneal dialysis; and third, a left donor kidney was transplanted into the right iliac fossa of the recipient. In our case, the allograft vein seemed to be slightly long and mildly twisted, as seen in digital subtraction angiography (DSA) images. Further, the patient experienced a rejection reaction (T cell- and antibody-mediated rejection) and was administered comprehensive antirejection treatment, including tacrolimus, mycophenolate mofetil, methylprednisolone, IVIG, and antihuman thymocyte immunoglobulin, overlapping his basic diseases, and all of which increased the risk of thrombosis.

TRVT is characterized by nonspecific symptoms, such as oliguria, anuria, distension, and pain in the transplanted kidney area. In severe cases, these symptoms can progress to ruptured bleeding of the transplanted kidney. The clinical manifestations of TRVT are nonspecific and difficult to distinguish from those of other urinary tract diseases or acute rejection^2,4,5^; therefore, a strong suspicion of TRVT is required for its detection.

Relying merely on clinical presentation and laboratory tests is insufficient for the diagnosis of TRVT. Further imaging is needed to assist in the diagnosis of patients with high clinical suspicion of TRVT. Angiography is the gold standard for the diagnosis of renal transplant vasculopathy; however, this procedure is invasive, involves ionizing radiation and contrast nephrotoxicity, and is often used only when interventional procedures are needed^1,4,14,15^. Ultrasonography is noninvasive, accessible, and free of ionizing radiation; accordingly, it is the preferred method for evaluating transplanted kidneys and can screen for the early detection of complications. In addition, it can also calculate the resistance index and artery indices to predict the prognosis of the uncomplicated transplanted kidney ^14,16^. Magnetic resonance imaging (MRI) provides excellent alternative criteria; it does not induce nephrotoxicity and possesses higher sensitivity relative to ultrasonography. However, it is not suitable for critical patients due to its associated time burden and lack of portability^17^. Compared to MRI, CTA has a shorter examination time and lower cost. Although contrast imaging is not required, there is still a risk of ionizing radiation and nephrotoxicity exposure^10,18^.

Anticoagulation is the first step in the treatment process. Based on anticoagulation, the most aggressive means are used to preserve the function of the transplanted kidney. Previous studies have reported that the treatment of venous thrombosis in allogeneic renal grafts mainly involves intravenous thrombolytic therapy and surgical thrombectomy or allograft exploration, with few attempts at local catheter-directed thrombolysis or percutaneous mechanical thrombectomy^2,4,9,15,18–20^. In our case, we used percutaneous pharmacomechanical thrombectomy plus transluminal balloon angioplasty, which is relatively well-established for treating lower-limb DVT. Therefore, we successfully adapted this method for the treatment of TRVT combined with ipsilateral lower-limb DVT. Compared to systematic thrombolysis, catheter-contact thrombolysis is more direct and effective and reduces the risk of bleeding associated with high-dose thrombolytic drugs. Successful emergency surgical thrombectomy has been reported in the early post-transplant period^5,18,19^. However, this procedure is highly invasive and increases the risk of anesthesia and infection; it is best used when mechanical stenosis must be corrected simultaneously due to technical transplantation complications. No extensive randomized controlled studies have assessed the therapeutic risks and efficacy of different treatment options.

The AngioJet Thrombus Removal System is a rheological mechanical thrombus suction device that works based on Bernoulli’s principle^4,21,22^. A common complication of the pharmacomechanical thrombectomy system is transient hemoglobinuria, which is caused by the release of free hemoglobin from ruptured red blood cells during thrombus aspiration^23^. Owing to the combination of renal insufficiency, postoperative CRRT was necessary in our case to maximize the clearance of free hemoglobin and safeguard renal function. Another complication of the pharmacomechanical thrombectomy system is pulmonary embolism caused by dislodgement of a thrombus by high-pressure water injection^10^. Partial capture of the dislodged thrombus by prepositioning the IVC retrievable filter was also a successful step of this procedure.

With respect to the timing of surgery, the probability of loss of graft kidney function due to venous thrombosis of the grafted kidney is exceptionally high, and effective emergency treatment after diagnosis is crucial. pharmacomechanical thrombectomy and Percutaneous balloon dilatation (PBA) treatment are effective in such cases. In our case, during follow-up, the patient’s renal function was restored, and no residual thrombosis or stenosis was observed. No severe complications associated with pharmacomechanical thrombectomy combined with PBA were identified during the procedure, postoperatively, or during the subsequent follow-up period.

In addition to early diagnosis and effective intervention, the development of preventive strategies is an integral part of this process, and technical factors play a significant role. To avoid excessive length and distortion of the transplanted renal vein, and to reduce repeated manipulation, the graft was made as ipsilateral as possible during surgery. To obtain correct anastomosis size and angle, the length of the allograft vein should be appropriately shortened^24–26^. Care should be taken to monitor blood volume when the patient is under anesthesia. After surgery, close monitoring of urine output, renal function, and ultrasound evaluation should be performed^27,28^. Furthermore, screening and matching of transplant donors, control of the recipient’s preoperative underlying diseases, improvement of general status, prompt detection and resolution of rejection reactions, selection of an appropriate antirejection regimen, and close monitoring are critical. It has also been suggested that low-dose aspirin and low-molecular-weight heparin may be beneficial in preventing RVT in high-risk patients, particularly in hypercoagulable patients or those who have received more than one renal artery graft^1,12,29^.

## Conclusions

In our case, pharmacomechanical mechanical thrombectomy plus transluminal balloon angioplasty was minimally invasive, safe, and effective for treating TRVT. However, further studies are needed to verify the efficacy and prognosis of different treatments for TRVT. After kidney transplantation, TRVT—an uncommon but serious complication—can develop, and in such cases, the donated kidney may be lost. The transplant team should develop a rigorous plan, coordinate with the multidisciplinary team to make an early and accurate diagnosis, and be proactive in resuscitating patients. Patients with high-risk factors should be closely followed up postoperatively. Lastly, due to the nonspecific clinical presentation of TRVT, we stress the importance of a strong index of suspicion.

### Ethics approval and consent to participate

All procedures were performed in accordance with the Declaration of Helsinki and approved by the Ethics Committee of Fujian Provincial Hospital, Fuzhou, China. All participants and legal guardians of the minors involved in the study provided written informed consent.

## Data Availability

The datasets analyzed for this study are available from the corresponding author upon reasonable request.
